# Effects of a Red Orange and Lemon Extract in Obese Diabetic Zucker Rats: Role of Nicotinamide Adenine Dinucleotide Phosphate Oxidase

**DOI:** 10.3390/jcm9051600

**Published:** 2020-05-25

**Authors:** Sara Damiano, Chiara Lauritano, Consiglia Longobardi, Emanuela Andretta, Ali Murat Elagoz, Paolo Rapisarda, Mattia Di Iorio, Salvatore Florio, Roberto Ciarcia

**Affiliations:** 1 Department of Veterinary Medicine and Animal Productions, University of Naples “Federico II”, 80137 Naples, Italy; emanuelaandretta94@gmail.com (E.A.); salvatore.florio@unina.it (S.F.); roberto.ciarcia@unina.it (R.C.); 2 Marine Biotechnology Department, Stazione Zoologica Anton Dohrn, 80121 Naples, Italy; chiara.lauritano@szn.it; 3 Department of Mental, Physical Health and Preventive Medicine, University of Campania “Luigi Vanvitelli”, 80138 Naples, Italy; consiglia.longobardi@unicampania.it; 4 Laboratory of Developmental Neurobiology, Department of Biology, Faculty of Science, KU Leuven, 3000 Leuven, Belgium; alimurat.elagoz@kuleuven.be; 5 Council for Agricultural Research and Economics (CREA), Research Centre for Olive, Citrus and Tree Fruit, 95024 Acireale, Italy; paolo.rapisarda@crea.gov.it; 6 Independent Researcher, 83100 Avellino, Italy; mattia.dri@hotmail.it

**Keywords:** kidney, diabetic nephropathy, anthocyanins, reactive oxygen species, NADPH oxidase, apoptosis

## Abstract

Diabetic nephropathy (DN) is the primary cause of end-stage renal disease, worldwide, and oxidative stress has been recognized as a key factor in the pathogenesis and progression of DN. Nicotinamide adenine dinucleotide phosphate (NADPH) oxidase has the most important contribution to reactive oxygen species generation during the development of DN. Bioactive compound use has emerged as a potential approach to reduce chronic renal failure. Therefore, a red orange and lemon extract (RLE) rich in anthocyanins was chosen in our study, to reduce the toxic renal effects during the development of DN in Zucker diabetic fatty rat (ZDF). RLE effects were examined daily for 24 weeks, through gavage, in ZDF rats treated with RLE (90 mg/kg). At the end of the experiment, ZDF rats treated with RLE showed a reduction of the diabetes-associated up-regulation of both NOX4 and the p47-phox and p22-phox subunits, and restored the BAX/BCL-2 ratio respect to ZDF rats. Furthermore, RLE was able to reduce the oxidative DNA damage measured in urine samples in ZDF rats. This study showed that RLE could prevent the renal damage induced by DN through its capacity to inhibit NOX4 and apoptosis mechanisms.

## 1. Introduction

Diabetes is a metabolic disorder that can cause chronic renal failure, including diabetic nephropathy (DN), where several human patients progressing to end-stage renal disease (ESRD) require dialysis or transplantation [1].

Interaction between environmental and genetic factors is the cause of diabetes development. Type 1 and type 2 diabetes are characterized by progressive end-stage renal disease, and apoptosis is probably the main form of cell death in both forms of the disease [2]. However, clinical definitions of disease often obscure different mechanistic subtypes. Type 1 diabetes is typically caused by an autoimmune assault against the B-cells, but the pathogenesis of type 2 diabetes is more variable, comprising different degrees of B-cell failure, relative to varying degrees of insulin resistance (which is often associated with obesity), and insulin secretion defects are major risk factors for type 2 diabetes [3]. A progressive decrease of the B-cell function leads to glucose intolerance, which is followed by type 2 diabetes, which inexorably aggravates with time [3]. Particularly, in patients with type-2 diabetes, insulin resistance is well-demonstrated and they might often need to take oral antidiabetic drug or insulin administration, to control the development of the pathology, as well as the DN [4].

The mechanisms responsible for the development of DN are complex and not yet clear. Several researchers have shown that oxidative stress and inflammation processes are implicated in the pathogenesis and progression of DN [5,6,7]. Oxidative stress combined with hyperglycemia can generate negative repercussions on the structure and function of the kidney, inducing, at the glomerular level, endothelial cell dysfunction, deposition of extracellular matrix, mesangial cell injury, dysfunction of the podocytes, increase in transforming growth factor β (TGF-β), cellular apoptosis, and microalbuminuria. In particular, pancreatic β-cell dysfunction and insulin resistance are observed in type 2 diabetes [3] and the DN is associated with an imbalance pro-oxidant/antioxidant and, in particular, with an overproduction of reactive oxygen species (ROS)/reactive nitrogen species (RNS), and a decrease in antioxidant enzymes (manganese superoxide dismutase-MnSOD, glutathione peroxidase-GPx, and catalase-CAT) [8]. The antioxidant-redox system primarily uses the nicotinamide adenine dinucleotide phosphate (NADPH) oxidase as a chemical reductor, which is mostly produced by the glucose-6-phosphate dehydrogenase [9].

Nicotinamide adenine dinucleotide phosphate (NADPH) oxidase (NOX) is involved in the regulatory processes of homeostasis and it appears to be the most important contributor to ROS generation in the kidney [10,11]. To date, the NOX family includes 5 members (Nox1, Nox2, Nox3, Nox4, and Nox5) but the most abundant NOX isoform in the renal system is NOX 4 [12,13]. In physiological conditions, NOXs activity is low, but in disease states, such as hypertension and diabetes, their levels increase, inducing the generation of reactive oxygen species (ROS). In particular, NOX isoforms transfer electrons across the biological membrane from NADPH, to reduce molecular oxygen to superoxide (O_2_^−^) [14]. It is well-demonstrated that NOX is composed of four major subunits—a plasma membrane gp91 phox subunit and a smaller p22-phox subunit; cytosolic p47-phox and p67-phox subunits; data in literature show a pivotal role of both p22-phox and p47-phox during the development of diabetes [15]. Moreover, the excessive renal activity of NADPH oxidase is considered to be one of the crucial factors for the progress of diabetic nephropathy and the inhibition of this enzyme is widely discussed as a promising new therapeutic strategy [16]. In fact, it is well-demonstrated by Katarzyna Winiarska et al. [17] that melatonin has a beneficial action against diabetic nephropathy, through attenuation of the excessive activity of Nox. For these reasons, NOX has been shown to be a potential target for pharmacological intervention during DN. In response to the overproduction of ROS during DN, several antioxidant molecules were tested, such as, tocotrienol, selenium, soy, catechins, omega-3 fatty acids, α-lipoic acid, and curcumin, showing an improvement of renal alterations by reversing the increased levels of ROS and activating antioxidant enzymes [18].

Therefore, the objective of the current work was to determine the role of a new red orange and lemon extract (RLE) that is naturally rich in anthocyanins (ANT), on the progression of DN. ANT are one class of flavonoid compounds, showing high anti-oxidant, anti-inflammatory activity, and anti-carcinogenesis properties [19]. The mechanism of action of ANT during the development of DN is not yet clear. Anjaneyulu and Chopra suggested a direct antioxidant action providing protection from DNA, protein, and lipid damage [20] and an indirect reduction of oxidative stress by activating specific detoxification enzymes, such as glutathione reductase, glutathione peroxidase, and glutathione S-transferase [21]. In our previous work [22], RLE was standardized in its relative levels of biologically active compounds and these levels, in different batches of the extract, were constant. RLE was obtained by properly mixing ANT and other polyphenols, recovered from red orange processing wastes, and eriocitrin and other flavanones, which were recovered from lemon peel. In this extract, the most abundant ANT was the cyanidin-3-glucoside (C3G). Part of the mechanism of action of C3G was attributed to the ability to chelate ions of bivalent metals, necessary to generate ROS through the Fenton reaction [23]. The other individual anthocyanin components represent a minor percentage. In fact, flavanones qualitative profile shows that the major flavanone compound is represented by eriocitrin, being present in a percentage equal to more than 10%, with respect to 80% of the total anthocyanins [22].

The present study was designed to evaluate if RLE (which has shown an inhibitory action on the development of DN in Zucker diabetic fatty rat (ZDF) [22]) could be considered to be a specific NOX inhibitor and could open new perspectives for the treatment of DN. The experiments were performed on young male ZDF rats, one of the most widely studied rodent models of diabetes type-2, considered to be especially useful for research focused on diabetic nephropathy [17,24]. It is considered a good experimental model that best recapitulates the pathogenesis and evolution of human T2DM and its cardiovascular complications [24]. We examined both gene and protein expressions of NADPH oxidase catalytic subunit, NOX4, and its regulatory subunits—p22-phox and p47-phox—through real-time PCR and western blot analysis. Moreover, DNA damage and apoptosis was investigated through the DNA Damage (8-OHdG) ELISA kit and gene and protein analysis of both pro-apoptotic protein BAX and anti-apoptotic BCL-2.

## 2. Experimental Section

### 2.1. Chemicals and Reagents

Rats were provided by Charles River Laboratories (Milan, Italy); the RLE was obtained by CREA-OFA (Acireale, Italy). Antibodies were purchased from Tema Ricerca s.r.l. (Bologna, Italy). Primers were synthetized by Sigma-Aldrich (Milan, Italy). All reagents were purchased from S.I.A.L s.r.l. (Rome, Italy).

### 2.2. Ethics Statement

This study was carried out in accordance with the recommendations in the Guide for the Care and Use of Laboratory Animals of the National Institutes of Health. All procedures complied with the current Italian and European law. The permit project number was 37-2016-PR.

### 2.3. Experimental Protocol

This study was conducted on 9 male control Zucker Fatty (ZF) rats and 18 male Zucker Diabetic Fatty (ZDF) rats, weighing 240–260 g. The experimental protocol began at 6 weeks of age in both ZF rats and ZDF rats. They were housed under constant environmental conditions (temperature 22 ± 2 °C, relative humidity of 40%–70%, artificial illumination on a 12-h light/dark cycle, and air exchange of 15 times/h); a standard diet was used ad libitum. Rats were divided into three groups and treated daily by gavage, from 6 weeks to 30 weeks of age, according to this scheme: ZF group (*n* = 9 control rats) received 1 mL of normal saline; ZDF Group (*n* = 9 ZDF rats) received 1 mL of normal saline; ZDF + RLE group (*n* = 9 ZDF rats) was treated with 90 mg/kg of RLE dissolved in 1 mL of normal saline. RLE dose administration was chosen according to previous experiments [25]. Normal saline solution was used for PH stability of the RLE extract. Animals were anesthetized with 2% isoflurane and the right femoral artery was then cannulated with polyethylene tubing connected to a blood pressure transducer (Powerlab, AD Instruments Inc., Colorado Springs, CO, USA), for monitoring the blood pressure (BP). The animals were sacrificed at 30 weeks of age, when DN was confirmed. At the end of all experiments, the animals underwent euthanasia by an overdose of 4% isoflurane (Isotec 4, Palermo, Italy), and blood samples were collected from the aorta and immediately centrifuged at 3950× *g* for 15 min at + 4 °C. Moreover, urine samples were taken from each group of animals housed in individual metabolic cages for 24 h, and finally, kidneys of each rat were rapidly removed in a cold room and immediately frozen at −80 °C.

### 2.4. RNA Extraction and Complementary DNA (cDNA) Synthesis

Four replicate tissues for each animal group (ZF, ZDF, and ZDF + RLE) were used for RNA extraction. Tissues were homogenized in 1 mL of the TRIZOL Reagent (Invitrogen, Thermo Fisher Scientific, Waltham, MA, USA), using TissueLyser (MM300, Retsch, Conquer Scientific, San Diego, CA, USA) and Tungsten Carbide Beads (3 mm) (Qiagen, Venlo, The Netherlands), for 5 min at 20.1 Hz, until all samples were completely homogenized. Total RNA was extracted using the TRIZOL manufacturer’s protocol and treated with DNase, as recommended by the manufacturer itself. RNA quantity was assessed by Nano-Drop (ND-1000 UV–Vis spectrophotometer; NanoDrop Technologies, Wilmington, NC, USA), monitoring the absorbance at 260 nm. The purity of each sample was assessed by monitoring the 260/280 nm and 260/230 nm ratios, using the same instrument (Both ratios were approximately 2.0). For RT-qPCR, 1000 ng for each sample were retrotranscribed into complementary DNA (cDNA) with the iScriptTM cDNA Synthesis Kit (BIORAD, Hercules, CA, USA), following the manufacturer’s instructions using the GeneAmp PCR System 9700 (Perkin Elmer, Waltham, MA, USA).

### 2.5. Selection of Gene of Interest and Primer Design

Five genes of interest (GOI) were selected—the pro-apoptotic protein BAX, the anti-apoptotic protein BCL-2, NADPH oxidase 4 (NOX4; Reactive oxygen species-generating enzyme found to be activated in diabetic rats), and NOX4 regulatory subunits p22-phox (p22) and p47-phox (p47); 18S was used as reference gene. Primers were designed using the software Primer3 v. 0.4.0 (http://frodo.wi.mit.edu/primer3/; Table 1) [26,27]. In addition, the software Gene Runner (V3.05, Hasting Software, Hastings, NY, USA) was used to predict the primer’s melting temperature (Tm) and to check if the primers formed dimers, hairpin, bulge, and internal loops. To determine the specificity of the amplification, the designed primer pairs were first tested in PCR, optimized in a GeneAmp PCR System 9700 (Perkin Elmer, Milan, Italy), according to the reaction conditions detailed in Lauritano et al. [28]. Amplified PCR products were then analyzed by 1.5% agarose gel electrophoresis in TBE buffer. Only PCR products that showed a single band on agarose gel were further considered for this study. The resulting single bands for each gene were excised from the gel and extracted with the GenEluteTM Gel Extraction Kit (Sigma-Aldrich, Saint Louis, MO, USA). Sequence reactions were obtained with the BigDye Terminator Cycle Sequencing technology (Applied Biosystems, Thermo Fisher Scientific, Waltham, MA, USA) in automation, using the Agencourt CleanSEQ Dye Terminator Removal Kit (Agencourt Bioscience, Beckman Coulter, Brea, CA, USA) and a Robotic Station Biomek FX (Beckman Coulter, Brea, CA, USA). Products were analyzed on an Automated Capillary Electrophoresis Sequencer 3730 DNA Analyzer (Applied Biosystems, Thermo Fisher Scientific, Waltham, MA, USA). The identity of each sequence was confirmed using the blastn function in the bioinformatics tool BLAST (Basic local alignment search tool; https://blast.ncbi.nlm.nih.gov/Blast.cgi).

### 2.6. Reverse Transcription-Quantitative Polymerase Chain Reaction (RT-qPCR)

RT-qPCR experiments were carried out in a Via7 real-time PCR system (Applied Biosystem, Thermo Fisher Scientific, Waltham, MA, USA). PCR volume of each sample was 10 µL with 5 µL of Fast Start SYBR Green Master Mix (Roche, Basilea, Switzerland), 0.7 pmol/µL for each oligo, and 1 µL of the cDNA template (at a dilution of 1:10). The following RT-qPCR thermal profile was used: 95 °C for 10 min, 40 cycles of 95 °C for 1 s, and 60 °C for 20 s, with a melting step between 60 °C and 95 °C [29]. The program was set to record every 0.5 °C. Single product amplification for each primer pair was ensured by creating a melting curve for each amplicon between 60 °C and 95 °C. Melting curve analysis indicated a single peak for each primer, which confirmed a gene-specific amplification and the absence of primer-dimers. The intra-assay variability was assessed by carrying out all RT-qPCR reactions in triplicates and by including three no-template negative controls (NTC) for each primer pair. Primer reaction efficiency (E) and correlation factor (R2) were determined by serial dilutions of cDNA (1:5, 1:10, 1:50, 1:100, and 1:500). Each oligonucleotide pair standard curve was plotted with the obtained dilution points by using the cycle threshold (Ct) value against the logarithm factor of each dilution and using the equation E = 10^−1^/slope. Primer efficiencies (E) ranged from 93% to 100%. For normalizing the GOI expression levels, *18S* was used as reference gene. The Excel-applet qGene [30,31] was used for the expression levels analysis.

### 2.7. Western Blot Analysis

NOX4, p22-phox, p47-phox, BCL-2, and BAX proteins expression were analyzed by western blot assay. Kidney tissues were homogenized in a lysis buffer (sucrose 0.3 M, imidazole 0.5 M, EDTA 0.5 M) with a protease inhibitor Mix (SERVA, Heidelberg, Germany). Mini-PROTEAN^®^ precast gel 4-12% (Bio-Rad, Milan, Italy) and Opti-Protein XL Applied Biological Materials Inc., Richmond, BC, Canada) as molecular weight marker were used. Trans-Blot^®^ Turbo PVDF membrane (Bio-Rad, Milan, Italy) was used to transfer proteins. The membranes were probed with primary antibodies—NOX4 (Rabbit polyclonal antibody, Cell Signaling, Leiden, The Netherlands); p47-phox and p22-phox (Rabbit polyclonal antibody, Santa Cruz Biotechnology, Heidelberg, Germany); BCL-2 (Rabbit polyclonal antibody, Cell Signaling, Leiden, The Netherlands); BAX (Rabbit polyclonal antibody, Cell Signaling, Leiden, The Netherlands), dilution 1:1000; and Tubulin (Mouse monoclonal antibody, Santa Cruz Biotechnology, Heidelberg, Germany); or Actin (Mouse monoclonal antibody, Santa Cruz Biotechnology, Heidelberg, Germany) as the housekeeping expression proteins, dilution 1:2000. Blots were incubated with HRP conjugated secondary antibodies (Santa Cruz Biotechnology, Heidelberg, Germany), according to the species of primary antibodies and were developed using the ECL substrate (Immobilon, Millipore, Milan, Italy). Signal intensity was quantified by the ChemiDoc™ Imaging System (Bio-Rad, Milan, Italy), with the Bio-Rad Quantity One^®^ software version 4.6.3. The results were expressed as arbitrary units.

### 2.8. Evaluation of DNA Damage

Oxidative DNA damage and oxidative stress were measured by the 8-OHdG ELISA kit from Stress Marq (Biosciences INC, Victoria, BC, Canada) in urine samples, in accordance with Varatharajan et al. protocols [32]. The 8-OHdG standards (0.5–80 ng mL^−1^) and 35–50 μL of urine were allowed to incubate separately for 1 h in a microtiter plate precoated with 8-OHdG. After the washing step of the primary antibodies, the secondary antibody was added and incubated for 1 h, followed by washing. The color developed by the addition of 3, 3′, 5, 5′—tetramethylbenzidine was measured at 450 nm, using a spectrophotometer Glomax Multi Detection System (Promega, Madison, WI, USA). Urinary 8-OHdG was expressed as total amount excreted in 2 h.

### 2.9. Statistical Analysis

The GraphPad InStat Version 3.00 for Windows 95 (GraphPad Software, San Diego, CA, USA) was used for statistical analysis. Statistically significant differences were evaluated by one-way analysis of variance (ANOVA), followed by Turkey’s post-test. The experiments were performed at least in triplicates. *p* < 0.05; *p* < 0.01; and *p* < 0.001 were considered statistically significant.

## 3. Results

### 3.1. Physiological Parameters: The Effect of RLE Treatment on Blood Pressure, and Food and Water Intake

Table 2 shows data on blood pressure (BP) expressed in mmHg, food intake expressed in g/day, and water intake expressed in mL/day of ZF, ZDF, and ZDF treated with RLE. BP of ZDF was not significantly increased compared to ZF and ZF + RLE rats (98.22 ± 9.6 mmHg in ZF, 104.10 ± 12.6 mmHg in ZDF rats, and 103.71 ± 6.9 mmHg in ZDF + RLE rats). Water intake was higher in the ZDF group with respect to ZF, but the RLE partially prevented this effect (14 ± 1.1 mL ZF, 58 ± 1.4 ZDF, 23 ± 1.7 ZDF + RLE). Food intake was 17 ± 0.8 ZF, 28 ± 2.0 ZDF, and ZDF + RLE was 21 ± 1.1, also showing a good effect of RLE on this parameter.

### 3.2. Gene Expression Results

#### 3.2.1. Nox 4, p22-phox, and p47-phox Gene Results

**Figure 1:**
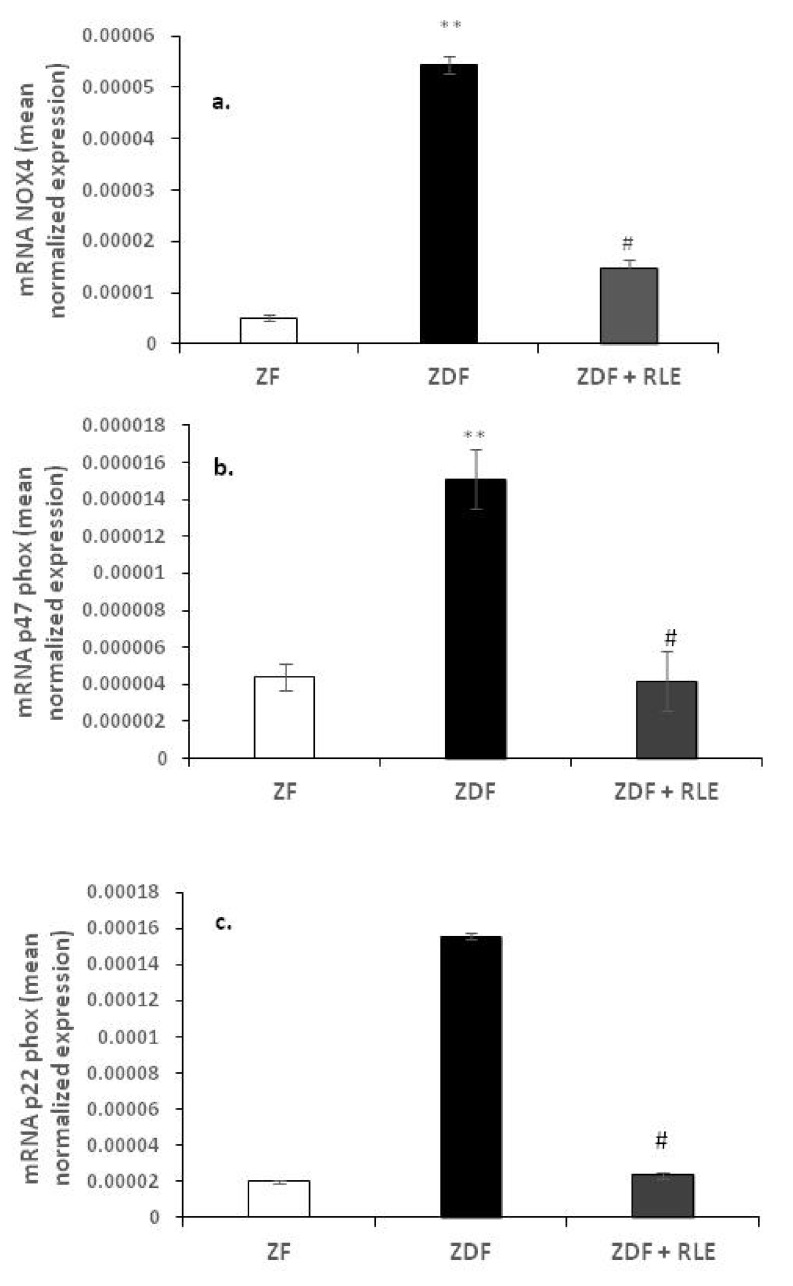
NOX4, p22-phox and p47-phox expression in Zucker Fatty (ZF), Zucker Diabetic Fatty (ZDF), and Zucker Diabetic Fatty + red orange and lemon extract (ZDF + RLE), after 24 weeks of treatment. (**a**) mRNA levels of NOX4 in ZF, ZDF, and ZDF + RLE; (**b**) mRNA levels of p47-phox in ZF, ZDF, and ZDF + RLE; and (**c**) mRNA levels of p22-phox in ZF, ZDF, and ZDF + RLE. Experiments were conducted in triplicates, and values are presented as mean normalized expression (MNE) normalized towards 18S expression (mean ± standard error) (** *p* < 0.01 versus ZF; # *p* < 0.05 versus ZDF).

Expression levels of NOX4 and its regulatory subunits, p22-phox and p47-phox, were investigated. Results showed that the expression levels of NOX4, p47-phox, and p22-phox, were significantly increased in diabetic ZDF rats, compared to ZF. In fact, NOX4 levels shifted from 5.45^−05^ ± 1.61^−06^ in the ZDF rats versus 5.11^−0.6^ ± 7.13^−0.7^ in ZF, ** *p* < 0.01 (Figure 1a), and the p47-phox levels shifted from 1.51^−05^ ± 5.91^−06^ in the ZDF rats versus 4.41^−0.6^ ± 7.74^−0.7^ in ZF, ** *p* < 0.01 (Figure 1b). Exposure of the ZDF rats to RLE induced a decreased expression of NOX4 (1.47^−05^ ± 1.61^−06^), # *p* < 0.05 (Figure 1a) and p47-phox (4.19^−06^ ± 1.51^−06^), # *p* < 0.05 (Figure 1b). Moreover, significant expression levels variations for regulatory subunit p22-phox was observed (1.55^−04^ ± 4.12^−06^ in ZDF rats versus 2.01^−0.5^ ± 5.26^−0.6^ in ZF, ** *p* < 0.01, 2.35^−0.5^ ± 3.32^−0.6^ in ZDF + RLE, # *p* < 0.05) (Figure 1c).

#### 3.2.2. BAX, BCL-2, and BAX/BCL2 Ratio Gene Results

**Figure 2:**
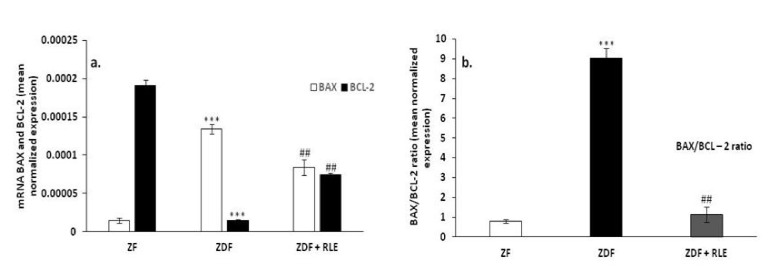
BAX, BCL-2, and BAX/BCL-2 ratio expression in the Zucker Fatty (ZF) (*n* = 4), Zucker Diabetic Fatty (ZDF) (*n* = 5), and Zucker Diabetic Fatty + red orange and lemon extract (ZDF + RLE) (*n* = 5), after 24 weeks of treatment. (**a**) mRNA levels of BAX and BCL-2 in ZF, ZDF, and ZDF + RLE; and (**b**) BAX/BCL-2 ratio in ZF, ZDF, and ZDF + RLE. Experiments were conducted in triplicates, and the values are presented as mean normalized expression (MNE), normalized towards 18S expression (mean ± standard error). *** *p* < 0.001 versus ZF, ## *p* < 0.01 versus ZDF).

Regarding genes involved in apoptosis regulation, results showed that the pro-apoptotic protein BAX was upregulated in diabetic rats ZDF (1.3^−0.4^ ± 6.32^−0.6^ versus 1.47^−0.5^ ± 3.17^−0.6^), *** *p* < 0.001 and downregulated in diabetic rats treated with RLE, with respect to ZDF (8.45^−0.5^ ± 1.01^−05^), ## *p* < 0.01, while the anti-apoptotic protein BCL-2 had the opposite trend (1.48^−05^ ± 6.91^−06^ in ZDF rats versus 1.91^−0.4^ ± 1.06^−0.7^ in ZF), *** *p* < 0.001 and 7.41−0.5 ± 3.24−0.5 in ZDF + RLE, ## *p* < 0.01 (Figure 2a). Moreover, results showed an increase in BAX/BCL-2 ratio in the ZDF rats (9.02 ± 0.5), with respect to the ZF (0.08 ± 0.1, *** *p* < 0.001), and the RLE treatment restored this value (1.14 ± 0.4, ### *p* < 0.001) (Figure 2b).

### 3.3. Western Blot Results

#### 3.3.1. NOX4, p22-phox, and p47-phox Proteins Results

**Figure 3:**
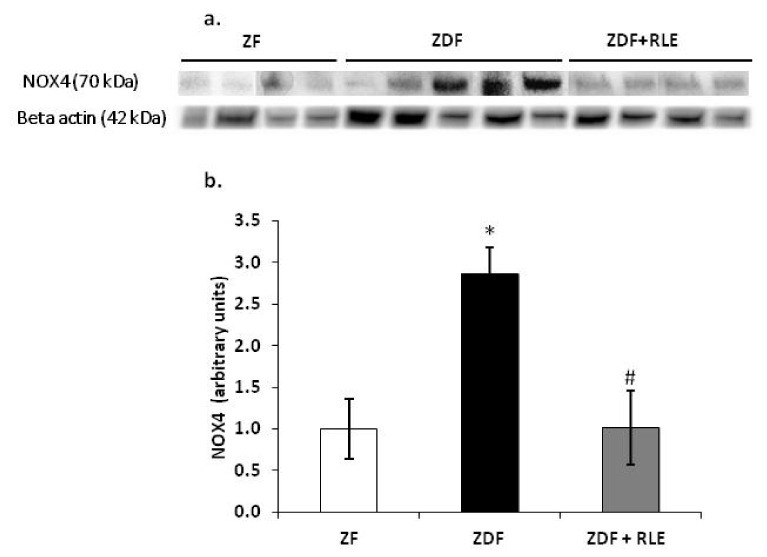
Effect of red orange and lemon extract (RLE) on the protein levels of the NOX4 in the Zucker Fatty (ZF) (*n* = 4), Zucker Diabetic Fatty ZDF (*n* = 5), and Zucker Diabetic Fatty + red orange and lemon extract (ZDF + RLE) (*n* = 4), after 24 weeks of treatment. (**a**) Representative western blot of NOX4 in ZF, ZDF, and ZDF-treated group. (**b**) The panel represents the densitometric analysis of NOX4. The experiments were conducted in triplicates, and the values were normalized towards actin. Densitometric analyses are expressed as arbitrary units. Data are shown as mean ± standard deviation (DS) and compared by ANOVA (* *p* < 0.05 versus ZF. # *p* < 0.05 versus ZDF).

**Figure 4:**
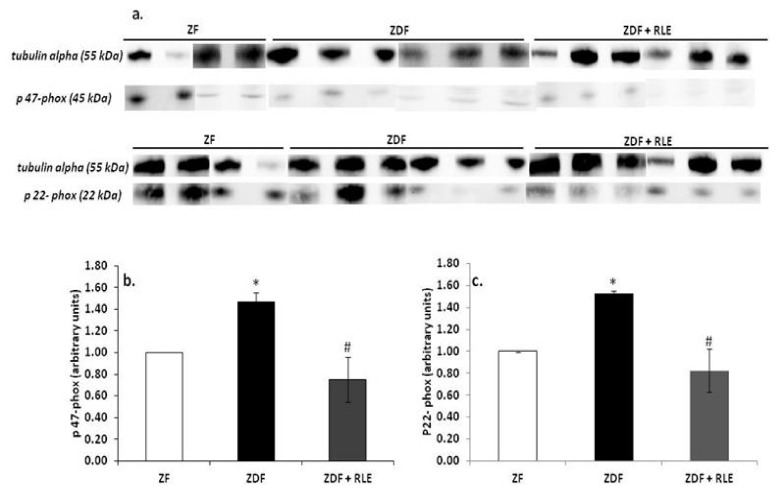
Effect of red orange and lemon extract (RLE) on the protein levels of p47-phox and p22-phox in the Zucker Fatty (ZF) (*n* = 4), Zucker Diabetic Fatty (ZDF) (*n* = 6), and Zucker Diabetic Fatty + red orange and lemon extract (ZDF + RLE) (*n* = 6), after 24 weeks of treatment. (**a**) Representative western blot of p47-phox and p22-phox in the ZF, ZDF, and ZDF treated group; (**b**) densitometric analysis of p47-phox; and (**c**) densitometric analysis of p22-phox. Experiments were conducted in triplicates, and the values were normalized towards the tubuline. Densitometric analyses were expressed as arbitrary units. Data are shown as mean ± standard deviation (DS) and were compared by ANOVA (* *p* < 0.05 versus ZF, # *p* < 0.05 versus ZDF).

Western blot analysis confirmed that NOX4 (Figure 3a,b), p47-phox (Figure 4a,b), and p22-phox (Figure 4a,c) proteins were significantly upregulated in the diabetic kidney of ZDF rats, at 30 weeks of age, with respect to the ZF animals. In fact, the NOX4 value was 1.00 ± 0.01 in ZF and 2.95 ± 0.8 in ZDF (* *p* < 0.05 respect to ZF) and the RLE treatment restored the ZDF value (1.01 ± 0.45, # *p* < 0.05).

The p47-phox value was 1.00 ± 0.01 in ZF and 1.47 ± 0.08 in ZDF (* *p* < 0.05 respect to ZF), and the p22-phox value was 1.00 ± 0.01 in ZF and 1.43 ± 0.3 in ZDF (* *p* < 0.05 respect to ZF). The RLE treatment for 24 weeks restored these values. In fact, p47-phox and p22-phox in ZDF + RLE were 0.75 ± 0.2 and 0.73 ± 0.2, respectively (# *p* < 0.5 with respect to ZDF).

#### 3.3.2. Ratio of the BAX/BCL-2 Expression Results

**Figure 5:**
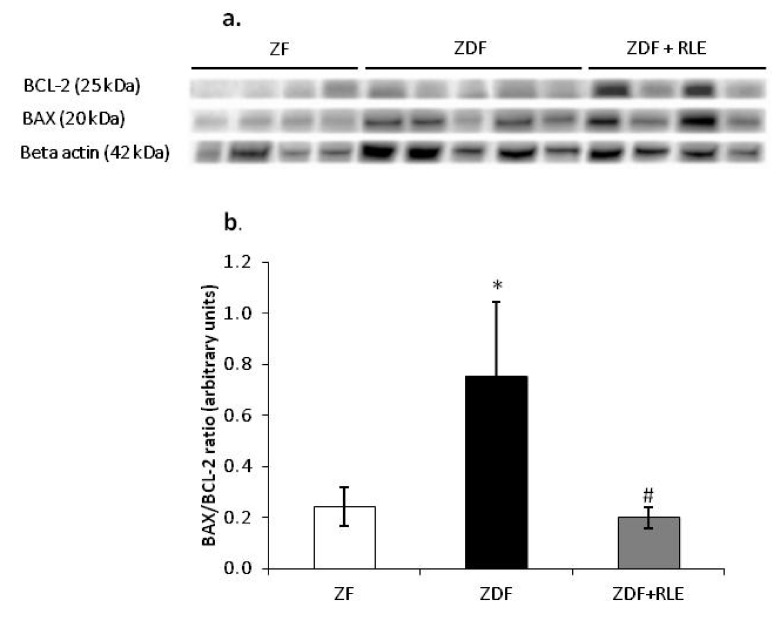
Effect of red orange and lemon extract (RLE) on the protein levels of the BAX/BCL-2 ratio in the Zucker Fatty (ZF) (*n* = 4), the Zucker Diabetic Fatty (ZDF) (*n* = 5), and the Zucker Diabetic Fatty + red orange and lemon extract (ZDF + RLE) (*n* = 4), after 24 weeks of treatment. (**a**) Representative western blot of BAX and BCL-2 in ZF, ZDF, and the ZDF-treated group; (**b**) densitometric analysis of the BAX/BCL-2 ratio. Experiments were conducted in triplicates, and the values were normalized towards the tubuline. Data are shown as mean ± standard deviation (DS) and were compared by ANOVA (* *p* < 0.05 versus ZF, # *p* < 0.05 versus ZDF).

With regards to the apoptosis regulation, the results showed that the apoptotic protein BAX/BCL-2 ratio was increased in diabetic ZDF rats, with respect to ZF (0.76 ± 0.29 ZDF versus 0.24 ± 0.08 ZF, respectively, * *p* < 0.05) and the RLE treatment restored this value in the ZDF rat (0.76 ± 0.29, # *p* < 0.05). It was interesting to note that these values were in accordance with the corresponding gene expressions (Figure 5a,b).

### 3.4. DNA Damage 8-Hydroxy-2-deoxy Guanosine (8-OHdG) Results

**Figure 6:**
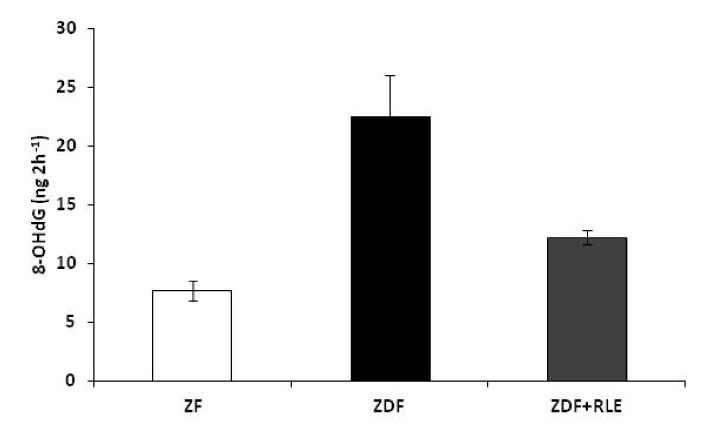
Effects of the red orange and lemon extract (RLE) on urinary 8-OHdG concentration, expressed in ng 2 h^−1^, in the Zucker Fatty (ZF), the Zucker Diabetic Fatty (ZDF), and the Zucker Diabetic Fatty + red orange and lemon Extract (ZDF + RLE), after 24 weeks of treatment. Data are shown as mean ± standard deviation (DS) (*n* = 6 for each group) and were compared by ANOVA (* *p* < 0.05 versus ZF, # *p* < 0.05 versus ZDF).

The oxidative DNA damage in the kidneys of ZF, ZDF, and ZDF + RLE rats was examined by measuring the 8-OHdG levels in urine samples. Urinary 8-OHdG levels were significantly higher in the ZDF rats with respect to the ZF control rats, at the end of the treatment (22.56 ± 3.5 ZDF ng 2 h^−1^ vs. 7.71 ± 0.8 ng 2 h^−1^), * *p* < 0.05. RLE oral intake in ZDF at the end of treatment significantly reduced urinary 8-OHdG levels (12.23 ± 0.6 ng 2 h^−1^, # *p* < 0.05), when compared to the ZDF rats (Figure 6).

## 4. Discussion

Oxidative stress has proven to be a key factor in triggering diabetic complications, such as nephropathy [33,34]. The present study was performed to investigate the antioxidant effects of the RLE (90 mg/kg) in a rat animal model of DN, the obese Diabetic Zucker rats. In a previous study, we have demonstrated the protective effect of RLE on glomerular filtration rate (GFR) and proximal tubule reabsorption and, although the exact molecular mechanism whereby anthocyanins act against DN is not fully understood, possible mechanisms could be the attenuation of high-sensitivity C-reactive protein and a reduced expression of sodium-dependent glucose transporter 1 [35,36]. Moreover, this effect was probably due to C3G, as has been demonstrated in previous works [37,38].

Furthermore, in our previous study we have demonstrated that the marked alteration of the glomerular filtration rate and of the proximal tubule reabsorption observed in the ZDF rats was prevented by oral administration of RLE, and we hypothesized that these events were correlated with its action on ROS productions [22]. In fact, in agreement with other research [39,40,41], in which the antioxidant protective effect on the diabetic nephropathy progression was demonstrated, we found that RLE restored the antioxidant pathways, in particular SOD, CAT, and GPx activities.

To confirming what we found in previous work, and in order to demonstrate that oxidative stress is involved in the pathogenesis and progression of DN, in this study, we also evaluated the oxidative DNA damage by measuring 8-OHdG levels in urine samples. 8-OHdG, an oxidized nucleoside of DNA, is the most frequently detected and studied DNA lesion. Upon DNA repair, 8-OHdG is excreted in the urine. Several studies have indicated that urinary 8-OHdG is not only a biomarker of generalized, cellular oxidative stress but might also be a risk factor for cancer, atherosclerosis, and diabetes [42]. In the present study, we observed a correlation between urinary concentrations of 8-OHdG and the severity of diabetic nephropathy. In fact, we observed elevated urinary 8-OHdG levels in ZDF rats, at 30 weeks of age when DN was confirmed, and a reduction of oxidative stress marker (8-OHdG) in the urine of ZDF rats treated with RLE (Figure 6). It was interesting to note that the highest concentration of 8-OHdG was associated with a lower GFR and proximal tubule reabsorption [22]. These data were in agreement with other studies in which the highest concentrations of 8-OHdG were reported in patients with chronic kidney disease or end-stage renal disease (ESRD), compared with healthy individuals [43,44], suggesting the potential use of 8-OHdG urinary as a biomarker of DN.

Moreover, in the present study, we explored RLE extract effects on NADPH oxidase renal expression and its subunits, p22-phox and p47-phox, because it is well-demonstrated that the NADPH oxidase is an important source of ROS production [31]. The NADPH oxidase is composed by membrane-bound subunits, such as p22-phox and gp91-phox, and cytosolic subunits, such as p47-phox, p40-phox, p67-phox, and Rac [45]. The increased expression of NADPH oxidase and its subunits is one of the mechanisms that contribute to increase oxidative stress in diabetic kidney [46,47] (Figure 1 and Figure 4). Here, we demonstrated a significant increase in renal expression of NOX4, p47-phox, and the p22-phox subunit in diabetic rats, through RT- PCR and western blotting experiments. Similarly, Sharma et al. found a significant p47-phox up-regulation in the hearts of type-1 diabetic rats [48], while Kassan et al. demonstrated that a high myogenic response in type-2 diabetic mice was a consequence of the increase in expression of p22-phox and down-regulation of p22-phox by siRNA, and the ROS scavenger restored the myogenic tone in diabetic mice [49]. A significant finding in this study was that the administration of RLE for 24 weeks reduced the diabetes-associated up-regulation of both NOX4 (Figure 1 and Figure 3) and of the p47-phox and p22-phox subunits (Figure 1 and Figure 4). We hypothesized that the inhibition of NOX4, the enzyme involved in the formation of O_2_^−^, was partially related to the antioxidant effect of RLE. RLE is rich in flavanones, in particular anthocyanins, and its high anti-oxidant, anti-inflammatory, and anti-carcinogenesis properties are well-demonstrated; whereas, at molecular level, it is known for its protective role against DNA, protein, and lipid damage [50]. In this study, we demonstrated that the protective role of RLE on the progression of diabetic nephropathy is, most likely, due to ANT’s ability to protect cells from O_2_^−^ by inhibiting NADPH oxidase activity.

Furthermore, to get further insights into the mechanisms for the protective effect of RLE intake during diabetes in kidney, we have also analyzed the involvement of apoptosis because it is well-demonstrated that oxidative stress can contribute to the regulation of this cell death event [51]. We found that the increase in NOX4 levels was associated with a significant growth of BAX/BCL-2 ratio in ZDF animals and this increase was completely prevented in ZDF rats treated with RLE (Figure 2 and Figure 5). Several authors showed data in accordance with our results. In fact, Lin et al. showed that quercetin-rich juice reduced apoptosis and pyroptosis formation in the kidney and pancreas of type II diabetic rats [52]. Wei et al. demonstrated that ANT inhibits high glucose-induced renal tubular cell apoptosis caused by oxidative stress in db/db mice [53]. Finally, food intake rich in natural compounds, such as flavonoid also contributed to restore the balanced activity of autophagy and apoptosis in the kidney of diabetic animals [50,51]. In particular, interesting data were related to the use of quercetin-rich juice or resveratrol, to improve the functionality of the organ [53,54,55].

In conclusion, we identified, according to several data in literature, the key role of NADPH oxidase in the development of DN complications, giving new insight into its mechanism of action, which contributes to vascular and functional diseases in type-2 diabetes. The capacity of RLE to prevent renal damage during the progression of the nephropathy through its capacity to inhibit NOX4 increase, might represent a novel approach to the treatment of diabetic nephropathy.

## Figures and Tables

**Table 1:** Gene names, primer forward (F) and reverse (R), amplicon size, oligo efficiencies (E) and correlation factors (R^2^), and GenBank accession numbers.

Gene Name	Primer F Primer R	Amplicon Size	E	R2	Acc. Number
*BAX*	ACAACAACATGGAGCTGCAG-CTTGGATCCAGACAAACAGCC	249	100	0.99	U32098.1
*BCL2*	GCCTTCTTTGAGTTCGGTGG-CTGAGCAGCGTCTTCAGAGA	221	100	0.99	L14680.1
*NOX4*	TCGGGTGGCTTGTTGAAGTA-GTCTGTGGGAAATGAGCTTGG	224	90	0.99	NM_053524
*18S*	AGAAACGGCTACCACATCCA-CCCTCCAATGGATCCTCGTT	158	93	0.99	NR_046237.1
*p22*	ATCAAGCAGCCACCTACCAA-GGGAGCAACACCTTGGAAAC	179	100	0.99	AJ295951.1
*p47*	TACGCTGCTGTTGAAGAGGA-GATGTCCCCTTTCCTGACCA	105	100	0.99	AY029167.1

**Table 2:** Effects of a red orange and lemon extract (RLE) on blood pressure (BP), food and water intake on Zucker Fatty (ZF) (*n* = 9), Zucker Diabetic Fatty (ZDF) (*n* = 9), and Zucker Diabetic Fatty plus red orange and lemon extract (ZDF + RLE) (*n* = 9) rats, after 24 weeks of treatment.

Group Rat	BP (mmHg)	Water Intake (mL/day)	Food Intake (g/day)
**ZF**	98.22 ± 9.6	14 ± 1.1	17 ± 0.8
**ZDF**	104.10 ± 12.6	58 ± 1.4 ***	28 ± 1.0 *
**ZDF + RLE**	103.71 ± 6.9	23 ± 1.7 *, ^###^	21 ± 1.1 ^#^

Data are expressed as mean ± standard deviation (DS), *n* = 9, * *p* < 0.05 and *** *p* <0.001 versus ZF, # *p* < 0.05 and ### *p* < 0.001 versus ZDF.
